# ViT-BiLSTM Multimodal Learning for Paediatric ADHD Recognition: Integrating Wearable Sensor Data with Clinical Profiles

**DOI:** 10.3390/s25206459

**Published:** 2025-10-18

**Authors:** Lin Wang, Guang Yang

**Affiliations:** 1 Department of Sports and Public Health, Faculty of Health and Life Sciences, University of Exeter, Exeter EX1 2LU, UK; 2 Department of Physical Education, Guilin University of Electronic Technology, Guilin 541004, China; 3 Department of Engineering Science, Institute of Biomedical Engineering, University of Oxford, Oxford OX3 7DQ, UK; guang.yang@eng.ox.ac.uk

**Keywords:** multimodal deep learning, ADHD prediction, Vision Transformer (ViT), accelerometer-based activity data, cross-attention fusion

## Abstract

**Highlights:**

**What are the main findings?**

- We propose a cross-attention multimodal framework that maps wrist-sensor time series to Gramian Angular Field (GAF) images, learns spatiotemporal representations with a Vision Transformer Bidirectional Long Short-Term Memory (ViT-BiLSTM) encoder, and uses cross-attention to align image and clinical representations while suppressing noise, thereby improving attention-deficit/hyperactivity disorder (ADHD) recognition.
- Compared with late concatenation and dual-stream baselines, cross-attention fusion achieves higher and more stable ADHD recognition accuracy across cross-validation folds.

**What is the implication of the main finding?**

- Integrating wearable data with lightweight clinical profiles enables scalable, objective ADHD screening in naturalistic, free-living settings.
- Using consumer wearables and brief clinical inputs, we can run low-cost ADHD screening in schools and communities, reducing referral delays and missed or wrong referrals; because devices are widely available and the workflow is light, it also scales in resource-limited settings and supports population-level screening for public-health planning.

**Abstract:**

ADHD classification has traditionally relied on accelerometer-derived tabular features, which summarise static activity but fail to capture spatial-temporal patterns, potentially limiting model performance. We developed a multimodal deep learning framework that transforms raw accelerometer signals into images and integrates them with clinical tabular data, enabling the joint exploration of dynamic activity patterns and static clinical characteristics. Data were collected from children aged 7–13 years, including accelerometer recordings from Apple Watches and clinical measures from standardised questionnaires. Deep learning models for image feature extraction and multiple fusion strategies were evaluated to identify the most effective representation and integration method. Our analyses indicated that combining activity images with clinical variables facilitated the classification of ADHD, with the ViT-BiLSTM model using cross-attention fusion achieving the highest performance. These findings suggest that multimodal learning can become a robust approach to ADHD classification by leveraging complementary information from activity dynamics and clinical data. Our framework and code will be made publicly available to support reproducibility and future research.

## 1. Introduction

### 1.1. Background

ADHD has recently gained increasing attention in research focused on early diagnosis and prevention, and it is one of the most common neurodevelopmental disorders in childhood [1,2,3]. It is characterised by symptoms of inattention, hyperactivity, and impulsivity that interfere with daily functioning and development [4,5]. Globally, an estimated 6–10% of children and adolescents meet ADHD diagnostic criteria [6,7]. Children with ADHD frequently encounter learning difficulties, impaired social communication, and elevated risks of mental health problems [8]. These challenges often extend into adolescence and adulthood, affecting future educational achievement, psychosocial, and overall quality of life [3,9,10].

However, traditional ADHD assessment methods can be broadly classified into clinical and non-clinical approaches. Clinical assessments, such as neuroimaging (e.g., magnetic resonance imaging), electroencephalography (EEG), and structured clinical interviews supplemented by standardised questionnaires, can provide relatively accurate diagnostic information and allow clinicians to evaluate symptom severity. However, these methods are costly, time-consuming, and require specialised equipment and professional expertise, which limits their accessibility and large-scale use [11,12]. Non-clinical approaches, by contrast, are mainly used for early screening and often rely on parent- or teacher-reported questionnaires. While these instruments are more feasible for population-level application, their accuracy may be compromised due to children’s limited cognitive abilities and the potential biases or inconsistencies in parent and teacher reports [13,14].

In contrast, monitoring physical activity using wearable sensors in naturalistic environments offers a unique opportunity to observe behavioural manifestations of ADHD in real time [11]. Children with ADHD may exhibit characteristic activity patterns, such as higher overall activity levels, more fragmented and irregular movement sequences, and difficulties in maintaining periods of low activity or stillness [15,16]. These distinctive patterns, which differ from those of typically developing children, can be objectively detected by accelerometer-based devices, enabling the identification of activity-derived biomarkers potentially linked to ADHD outcomes [11,17].

**Figure 1:**
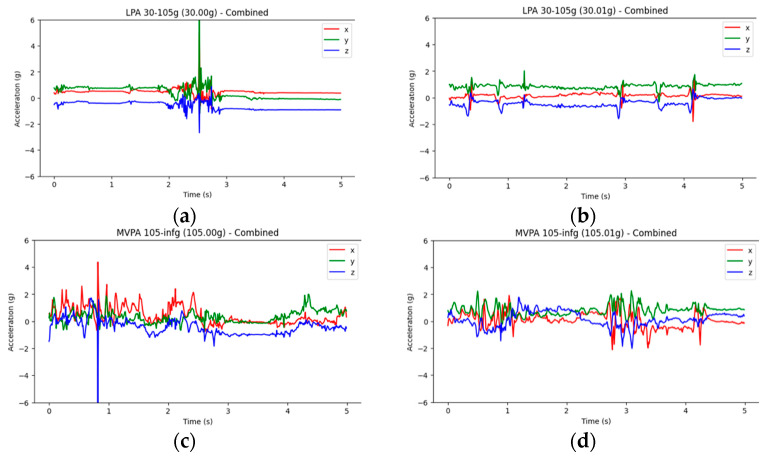
Gravity acceleration curve of physical activity: (**a**,**b**) represent more light physical activity, while (**c**,**d**) represent more moderate physical activity, both under similar gravity acceleration. This figure shows the acceleration data collected by a three-axis sensor (e.g., an Apple Watch worn on the dominant hand), the *x*-axis points in the front-back direction along the wrist, the *y*-axis points in the side-to-side direction (perpendicular to the arm), and the *z*-axis points upward, perpendicular to the surface of the device (toward the hand).

Although Previous studies have established a relationship between daily physical activity and ADHD symptoms in children [18,19]. Existing machine learning applications for predicting ADHD in children often rely on low-dimensional data derived from physical activity, such as average activity intensity or time spent, commonly represented in tabular formats [20,21]. This approach limits their ability to offer deeper insights into activity behaviours and ADHD prediction accuracy. In contrast, high-dimensional, image-based data can simultaneously present richer information [22], such as intensity, peaks, and variation patterns, which may significantly enhance model performance. For example, as shown in Figure 1, people with similar cumulative activity levels may exhibit distinctly different movement patterns. Such temporal patterns could be critical in understanding ADHD-related hyperactivity and attention fluctuations.

Many fields naturally involve multimodal data, such as medicine, where medical images and clinical data are combined for diagnosis, and sentiment analysis, which incorporates diverse factors. Integrating these data types into models helps create more robust representations and further improves predictive performance [23]. Also, attention-based spatiotemporal graph neural networks have shown strong performance in trajectory prediction tasks by effectively capturing both interactions and temporal dependencies [24], which further supports the use of attention mechanisms in multimodal temporal modelling for clinical applications.

Conventional signal acquisition typically relies on machine-based laboratory systems, which are often expensive, non-portable, and not feasible for long-term monitoring in naturalistic settings. Recently, portable consumer devices have become increasingly popular for collecting data on personal activities. Among them, the Apple Watch has been widely adopted due to its accessibility and convenience. Additionally, although the Apple Watch is a consumer-grade wearable, previous validation studies have demonstrated acceptable reliability in capturing accelerometry signals and estimating physical activity intensity levels [25,26]. Moreover, its wrist-worn form factor is child-friendly, thereby facilitating naturalistic data collection in free-living environments without disrupting children’s everyday routines. Specifically, the Apple Watch enables data extraction in real time, which increases the feasibility of continuous monitoring and supports the timely assessment of behavioural patterns relevant to ADHD.

### 1.2. Related Work

Existing studies on ADHD prediction predominantly use sensor-measured physical activity data, with machine learning models such as support vector machines (SVMs) [27,28], random forest (RF) [3,11,29], XGBoost (XGB) [30], and LightGBM (LGB) [11] being widely applied. Among them, SVM reached 94% accuracy [27], RF achieved 87% accuracy [29], and more advanced techniques such as elastic net and ridge regression achieved an area under the curve (AUC) of 89% [31]. Some studies have incorporated additional clinical or psychological variables to enhance prediction (e.g., emotion-related features) [11]. Their integration strategy remains simplistic, often treating these variables independently from the activity data.

One notable exception is the CNN-based approach by Muñoz-Organero et al. (2018) [32], which utilised image-based representations of physical activity, achieving a 93% accuracy. Despite these encouraging results, several methodological limitations persist across this body of work. First, most studies rely on low-dimensional tabular features derived from summary statistics (e.g., mean or standard deviation of activity), which are inadequate in capturing the temporal and dynamic nature of physical activity patterns, an aspect particularly salient to ADHD symptomatology. These approaches often compress rich time-series signals into static features, potentially discarding temporal cues indicative of hyperactivity, impulsivity, or inattention. In other words, by treating dynamic behaviours as static entities, existing models may not fully reflect behavioural dysregulation patterns that characterise ADHD.

Previous studies have not leveraged multimodal frameworks that jointly integrate sensor-derived time-series signals with demographic or clinical variables for ADHD prediction (Table 1). Moreover, comparative evaluations of alternative data fusion strategies and their potential to enhance model performance remain largely unexplored. The only reported study re-encoded activity time series as image representations and applied a deep learning model for classifying ADHD. Building on these, the present study proposes a multimodal deep learning framework (VB-Multimodal) that integrates clinical variables with image-based representations of physical activity. The VB-Multimodal was based on recent advances in multimodal fusion [33,34] and applied the ViT-BiLSTM memory to capture both spatial–temporal movement patterns (through GAF-transformed activity images) [20,21,35]; then, the model fuses individual-level clinical characteristics (e.g., age, sex, body mass index (BMI), symptom scores) with the image features using a cross-attention strategy. Finally, the model explores various fusion mechanisms to enable dynamic interactions between modalities and enhance the integration of behavioural and clinical features, providing a novel approach to ADHD prediction from a multimodal perspective.

### 1.3. New Contributions

In this work, we propose a multimodal framework for ADHD classification that integrates physical activity images with clinical tabular data. The main contributions are summarised as follows:

Novel multimodal architecture: We re-encode wrist-accelerometer time series as GAF images to expose richer activity signatures, learn spatiotemporal features with a ViT-BiLSTM branch, and fuse them with clinical profiles via cross-attention, resulting in improved accuracy of ADHD recognition.

Extensive experimental evaluation: We conduct systematic comparisons of image encoders and fusion strategies, demonstrating that cross-attention fusion consistently outperforms alternative approaches and achieves state-of-the-art performance.

Data collection and open access: We establish and release a multimodal dataset containing both GAF-transformed accelerometer images and structured clinical variables, enabling investigation of ADHD-related behavioural patterns in free-living conditions.

## 2. Materials and Methods

### 2.1. Participants and Data Collection

This study used a dataset collected by our research team, which has been previously published. Ethical approval for the data collection was obtained in advance (Approval No. 202309801). The dataset has since been made publicly available and described in detail in our prior publication [36]. The study employed an open dataset with 50 participants, an average age of 9.16 years (SD = 1.68, range: 7–13), and an average BMI of 16.57 (SD = 2.69, range: 11.7–22.8). The sample consisted of 29 males and 21 females.

The Apple Watch Series 7 (Apple Inc., Cupertino, CA, USA) was selected as the primary accelerometry device, as recent validation studies have demonstrated its acceptable reliability and validity for measuring physical activity in research settings [25,26]. Data acquisition was conducted using the SensorLog application (version 5.2, Bernd Thomas, Berlin, Germany), a widely adopted tool for recording raw accelerometer signals [26]. For this study, acceleration data were sampled at 100 Hz, a frequency consistent with recent paediatric studies and sufficient to capture detailed and fine-grained movement characteristics [37].

### 2.2. Data Preprocessing

The preprocessed data involved preparing two main types of input: (1) time-series physical activity data collected via wearable accelerometers, and (2) tabular features comprising clinical and socio-demographic information (e.g., age, sex, BMI). Activity data from 50 participants were processed to generate GAF images, resulting in a total of 5542 images. These were split into 2722 for training, 941 for validation, and 1879 for testing, ensuring a balanced distribution for robust model evaluation. This integration permits a holistic analysis of activity data and clinical features to predict ADHD symptoms.

#### 2.2.1. Physical Activity Acceleration to Generate an Image

The process of generating images from activity acceleration begins with the calculation of the Euclidean Norm Minus One (ENMO).

(1) $$ENMO=\sqrt{x^{2}+y^{2}+z^{2}}-1g\;$$

ENMO is a metric used to quantify the magnitude of acceleration while accounting for gravitational acceleration (1 g = 9.81 m/s), which represents the gravity accumulation. It is calculated as the Euclidean norm (length) of the three-dimensional acceleration vector (x, y, z) minus 1 g [38].

Once the ENMO values are calculated for each point, they are normalised and transformed into a polar coordinate system using the GAF method. In this transformation, each normalised acceleration value is represented as a polar angle and radius, which are then converted into pixel values to create GAF images [39]. To ensure consistency across all samples, the signal sequence $\left(v_{t}\right)$ was normalised to the range [−1, 1]. The normalisation formula is as follows:

(2) $$\tilde{v_{t}}\;=\;\frac{v_{t}\;-\;\min\left(v\right)}{\max\left(v\right)\;-\;\min\left(v\right)}\;\cdot \;2\;-\;1,\;\forall \;t$$

where $v_{t}$ is the original signal value at time $t,\min\left(v\right)$ and $\max\left(v\right)$ represent the minimum and maximum values of the signal, respectively, and $\tilde{v_{t}}$ is the normalised signal value.

The normalised signal $\tilde{v_{t}}$ is mapped into a polar coordinate system, representing the signal in terms of angles and radii.

$$Angle\left(\theta_{t}\right)$$

Each normalised signal value is converted into an angular value using the inverse cosine function:

(3) $$\theta_{t}=\arccos\left(\tilde{v_{t}}\right),\;-1\leq \tilde{v_{t}}\leq 1$$

The angle encodes the relative amplitude of the signal.

Radius (r_t_)

(4) $$r_{t}=\frac{t}{T},\;t=1,2,\ldots ,T$$

The radius represents the normalised time index, preserving the sequential nature of the time series, where T is the total length of the signal sequence.

Using the angular values $\theta_{t}$ from the polar representation, the GAF matrix is constructed to capture the temporal dependencies of the time series. The matrix elements are defined as follows:

(5) $$G\left[i,j\right]=\cos\left(\theta_{i}+\theta_{j}\right),\;i,j=1,2,\ldots ,T$$

where G[i,j] represents the cosine of the sum of the angles at time points i and j. This formula encodes both global and local temporal features of the signal. To facilitate visualisation, the GAF matrix values were normalised to the range [0, 1].

#### 2.2.2. Tabular Data

The tabular dataset consisted of both socio-demographic and clinical variables collected for each participant. Socio-demographic information included sex, age, and BMI, as well as indicators of health status, financial situation, dietary habits, parental education, and physical fitness. Categorical variables such as sex were numerically encoded (e.g., male = 0, female = 1) for model compatibility. ADHD symptoms were assessed using the 26-item Swanson, Nolan, and Pelham Questionnaire (SNAP-IV), a widely used parent- and teacher-rated instrument derived from DSM criteria. The scale consists of three domains: inattention (9 items), hyperactivity/impulsivity (9 items), and oppositional defiant behaviours (8 items). Each item is rated on a four-point Likert scale ranging from 0 (not at all) to 3 (very much), with higher scores indicating greater symptom severity. Subscale scores can be calculated for each domain, while the total SNAP-IV score is obtained by summing all 26 items to reflect overall ADHD symptom burden. The SNAP-IV has demonstrated good internal consistency and construct validity across diverse child and adolescent populations and has been applied extensively in both clinical and research settings [40]. To standardise the total score across participants, a Z-score transformation was first applied using the sample mean and standard deviation. This Z-score was then converted into a T-score using the following formula: T = (Z × 10) + 50. Participants with a T-score of 55 or higher were classified as exhibiting ADHD symptoms, while those below this threshold were categorised as non-ADHD [41].

#### 2.2.3. Preprocessing of Tabular Variables

To ensure data quality and compatibility with the multimodal learning framework, several preprocessing steps were applied to the tabular dataset. Missing values in numeric variables were imputed using the sample mean, while categorical variables were imputed using the most frequent category. All categorical variables (e.g., sex) were numerically encoded (male = 0, female = 1), a standard practice in clinical and machine learning applications. Consistency checks were performed to verify that no spurious categories or extreme outliers were present. Finally, clinical scale scores (SNAP-IV subscales and total score) were aggregated following validated scoring protocols, and the derived T-scores were used to generate the binary ADHD label, ensuring harmonisation of clinical data for integration with sensor-derived features.

### 2.3. Imaging Architectures

We adopt the Vision Transformer (ViT) as the backbone for image-based feature extraction. The input to the model is not a single static image but a sequence of activity images over time (i.e., 2D + temporal dimension), each resized to 224 × 224 pixels. To repurpose the ViT for feature encoding, we remove its classification head and extract the output from the final encoder layer, obtaining a 768-dimensional feature vector per frame. These feature vectors are subsequently passed into a BiLSTM network, configured with an input dimension of 768, a hidden size of 128, and two stacked layers. The bidirectional structure enables the model to capture sequential dependencies in both temporal directions, generating a 256-dimensional representation per image sequence. To enable multimodal learning, the resulting image embeddings are integrated with structured clinical features via attention-based cross-modal interaction. This unified representation is then used for downstream ADHD classification.

### 2.4. VB-Multimodal Architectures

**Figure 2:**
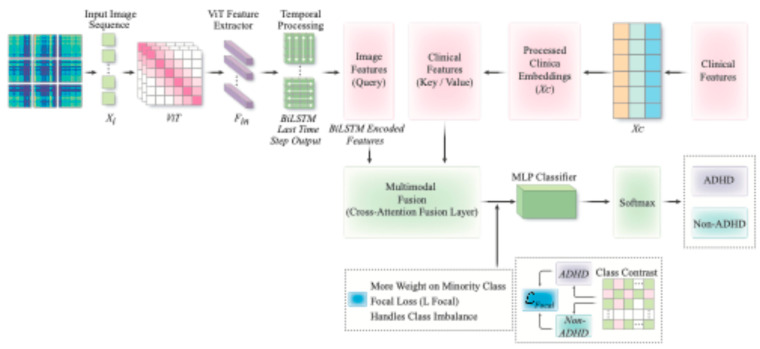
VB-Multimodal ADHD classification framework. The arrows in the figure represent the flow of information within the model. The pink blocks represent the multimodal information inputs, while the green blocks represent the fusion strategies.

Figure 2 illustrates the overall architecture of the proposed multimodal classification model, which integrates image-based activity features and clinical data using a cross-attention fusion layer. The ViT extracts spatial features from GAF images, while the BiLSTM captures temporal dependencies. These features are combined with processed clinical embeddings for the classification of ADHD. Cross-Attention Fusion employs a cross-modal attention mechanism, where tabular features function as the Query (Q), and image features serve as the Key (K) and Value (V). The fused features are computed using multi-head attention as follows:

(6) $$F_{\mathrm{fused}}=\mathrm{softmax}\left(\frac{Q\cdot K^{⊤}}{\sqrt{d_{k}}}\right)\cdot V$$

where Q = W_Q_ ⋅ F_tabular_ ∈ R^1×128^, K = WK ⋅ F_image_ ∈ R^1×128^, V = WV ⋅ F_image_ ∈ R^1×128^, d_k_ = 128, which is the dimensionality of the Key. And the fused features (F_fused_) are passed through a shared fully connected layer for classification, y = softmax (W_fused_⋅F_fused_ + b), where F_fused_ ∈ R^128^, W_fused_ ∈ R^2×128^, which maps the hidden dimension (128) to the number of classes (2, ADHD and Non-ADHD), b ∈ R^2^, representing the bias term.

In this multimodal framework, each input sample consists of (i) a sequence of five 2D activity images generated from raw acceleration (size: 5 × 3 × 224 × 224), and (ii) a clinical feature vector containing demographic-related tabular variables. The image sequence is first passed through a ViT, whose classification head is removed, yielding a 768-dimensional feature for each frame. These features are then fed into a BiLSTM layer to model temporal dependencies across the sequence, resulting in a spatiotemporal representation F_image_ ∈ R^1×768^ per sample.

The clinical variables are preprocessed (standardised) and directly used as input vectors, denoted F_tabular_ ∈ R^1×d^, where d is the number of selected clinical features. Before entering the cross-attention fusion module, the tabular vector is linearly projected into the Query space using W_Q_ ∈ R^128×d^. The image-based features are then projected to the Key and Value spaces using separate linear mappings.

### 2.5. Experimental Setup

To ensure robust evaluation and prevent information leakage across subsets, the dataset was partitioned at the participant level into non-overlapping groups: 55% for training, 15% for validation, and 30% for testing. Stratified sampling was applied to preserve the distribution of ADHD and non-ADHD cases across all subsets. This design ensured that no participant appeared in more than one subset, thereby reducing the risk of overfitting and enhancing the validity of model generalisation.

The model was trained for 20 epochs using a learning rate of 1 × 10^−4^, a batch size of 8, and a weight decay of 1 × 10^−5^. To further mitigate overfitting and assess the stability of model performance, we adopted 10-fold stratified cross-validation, which guarantees that each fold maintains the class distribution while providing multiple training–validation splits. Additionally, Focal Loss with class weights was employed to address class imbalance. A dropout rate of 0.5 was applied to the fully connected layers. The Vision Transformer backbone employed 12 attention heads, following its standard configuration. No explicit scheduler for learning rates was used beyond the fixed initial learning rate. All experiments were initialised with a fixed random 42 seed to ensure reproducibility. All experiments were conducted on an NVIDIA Tesla V100 GPU (Santa Clara, CA, USA) in UK with 32 GB of memory.

### 2.6. Model Assessments

To provide a more comprehensive evaluation of model performance, we adopted multiple standard metrics that reflect various aspects of predictive effectiveness, especially in the presence of class imbalance [42,43,44]. These include accuracy, precision, recall, F1 score, area under the ROC curve (AUC-ROC), and the Matthews correlation coefficient (MCC). Formal definitions and relevant references are provided below. True positive (TP), true negative (TN), false positive (FP), and false negative (FN) represent the four possible outcomes of a binary classification model, capturing correct and incorrect predictions for both positive and negative classes (Formulas (7)–(12)).

(7) $$\mathrm{Accuracy}=\frac{TP+TN}{TP+TN+FP+FN}$$

Accuracy measures the proportion of correctly classified instances among the total number of samples, providing an overall estimate of model correctness.

(8) $$Precision=\frac{TP}{TP+FP}$$

Precision evaluates the proportion of true positives among all predicted positives, reflecting how reliable the positive predictions are.

(9) $$Recall=\frac{TP}{TP+FN}$$

Recall measures the model’s ability to identify all relevant positive cases, indicating how well it avoids false negatives.

(10) $$F_{1}=\frac{2PR}{P+R}$$

The F_1_ Score is the harmonic mean of precision (P)and recall (R), and provides a single metric that balances both. It is particularly useful for imbalanced datasets.

Higher values of accuracy, precision, recall, and F_1_ score (closer to 1.0) indicate better model performance and stronger predictive reliability [42].

$$AUC=\int_{0}^{1}TPR\left(FPR^{-1}\left(x\right)\right)\;dx,\;$$

(11) $$where\;TPR=\frac{TP}{TP+FN},\;FPR=\frac{FP}{FP+TN}\;$$

ROC-AUC quantifies the area under the ROC curve, which plots the true positive rate against the false positive rate at different thresholds, offering a threshold-independent evaluation of classification performance. AUC values range from 0.5 to 1.0, where 0.5 denotes random guessing and 1.0 denotes perfect discrimination between classes [45].

(12) $$MCC=\frac{TP\times TN-FP\times FN}{\sqrt{\left(TP+FP\right)\left(TP+FN\right)\left(TN+FP\right)\left(TN+FN\right)}}$$

MCC is a correlation coefficient between the observed and predicted classifications, which performs well even in the presence of class imbalance, yielding a value between −1 and +1, where +1 indicates perfect prediction, 0 corresponds to random performance, and −1 represents complete disagreement between predictions and true labels [46].

## 3. Results

### 3.1. Participants’ Characteristics

Table 2 characterises the cohort used for model development (N = 50; 29 male, 21 female). Baseline demographics are comparable across sex: age (9.16 ± 1.68 y; *p* = 0.95) and BMI (16.57 ± 2.69 kg/m^2^; *p* = 0.24) show no significant differences. Grade levels are concentrated in Grades 1–4 (≈90%; overall *p* = 0.61). Socioeconomic indicators are skewed toward favourable profiles (health good: 86%; diet good: 70%; parental education moderate: 80%), with no material sex differences (e.g., parental education *p* = 0.940). Categorical variables are reported as n (%), continuous as mean ± SD; group comparisons use Welch’s *t*-tests (continuous) and χ^2^/Fisher tests (categorical).

ADHD-related symptomatology exhibits clear sex separation: males score higher than females on inattention (16.93 ± 4.02 vs. 14.14 ± 3.76; *p* = 0.01), hyperactivity/impulsivity (14.72 ± 3.39 vs. 12.52 ± 3.09; *p* = 0.02), oppositional (14.24 ± 2.84 vs. 12.71 ± 1.95; *p* = 0.02), and total SNAP-IV (45.69 ± 9.23 vs. 39.24 ± 7.72; *p* = 0.01). Using the SNAP-IV T-score threshold, overall ADHD prevalence is 26% (13/50), higher in males (37.9%) than in females (9.5%; *p* = 0.02).

### 3.2. VB-Multimodal Results

This study compares the performance of different machine learning and deep learning models within the same multimodal ADHD classification framework, evaluates various multimodal fusion strategies using the ViT-BiLSTM model, and validates the model’s robustness through 10-fold cross-validation.

Table 3 summarises the average performance of the multimodal model with the ViT-BiLSTM model across the ten folds. The results show consistently strong performance across all metrics. The model outperforms all other models, achieving the highest accuracy (0.97), precision (0.97), recall (0.97), F1 score (0.97), AUC (0.99), and MCC (0.93).

Previous studies using tabular physical activity data reported accuracy: Faedda et al. (2016) achieved 83% with SVM [27], Duda et al. (2017) reached an AUC of 89% using regression models [31], and Rahman (2024) obtained the accuracy of 87% [29] and Jiang et al. (2024) improved accuracy to 93% by incorporating emotion-related features [30]. Muñoz-Organero et al. (2018) achieved 93% with a CNN but relied on fixed coordinate transformations, potentially losing temporal features [32].

Our ViT-BiLSTM model surpasses prior methods, achieving 97% accuracy-4% to 14% higher. This improvement stems from capturing fine-grained movement patterns and integrating clinical data with high-dimensional activity representations, highlighting the advantages of a multimodal deep learning approach.

Among all methods, cross-attention achieves the best performance, with the highest accuracy (0.97), precision (0.97), recall (0.97), F1 score (0.97), and AUC (0.99), along with a strong MCC of 0.93, indicating its effectiveness in integrating image and tabular data. Compared to other fusion methods, it captures complex relationships between activity images and tabular data better (Table 4).

To ensure the reliability of the results, we conducted 10-fold cross-validation, and the validation results demonstrate the model’s stability. The mean AUC achieved was 0.99 ± 0.004, indicating a high discriminative ability. The mean MCC was 0.92 ± 0.06, reflecting strong agreement between predictions and true labels. The mean F1 score was 0.96 ± 0.03, with a mean recall of 0.96 ± 0.03 and a mean precision of 0.96 ± 0.02, confirming the model’s robustness and consistent performance across different validation folds.

**Figure 3:**
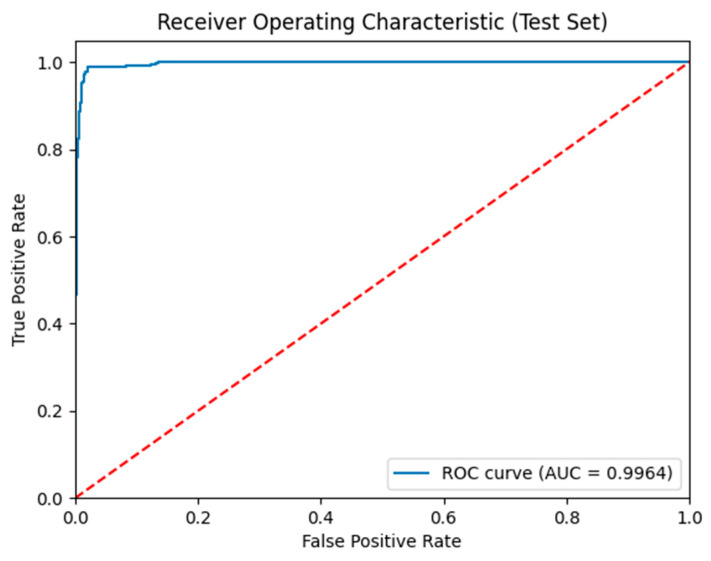
ROC for model performance evaluation. The red line represents the diagonal reference line, indicating a random classifier. The blue line shows the balance between sensitivity and specificity; the closer the curve is to the upper left corner, the better the model distinguishes between the two classes.

Figure 3 shows the ROC for the test set. The solid blue line represents the ROC curve of the model, showing a better classification ability, as it remains close to the top-left corner. The AUC is 0.99, indicating excellent discriminative power, with the model achieving high sensitivity and specificity in distinguishing between ADHD and non-ADHD cases.

## 4. Discussion

The present study introduces a multimodal pipeline that encodes raw wrist accelerometry into GAF images and fuses these spatial-temporal representations with structured clinical variables via a cross-attention module on top of a ViT-BiLSTM encoder. This design yields high discrimination (AUC ≈ 0.99; accuracy ≈ 0.97) and stable cross-validated performance. Recent comparative studies report that ViT can outperform CNNs on several medical classification tasks when data augmentation and token regularisation are adequate [47]. This advantage is particularly relevant for physical activity data, where GAF images encode long-range temporal dependencies; ViT patches and self-attention enable effective modelling of these global structures [20,35], while BiLSTM aggregates inter-image temporal context [48]. Beyond this, the cross-attention mechanism further augments the ViT backbone by allowing activity-derived image features to dynamically interact with structured clinical variables, thereby enhancing multimodal synergy and boosting overall classification performance [49,50].

Recent studies in multimodal learning consistently report that cross-attention fusion outperforms traditional concatenation or summation strategies by enabling more fine-grained interactions between modalities [51,52,53]. Although no prior multimodal frameworks have applied cross-attention specifically for ADHD prediction, its effectiveness has been consistently demonstrated in related biomedical and multimodal tasks. For example, Tang et al. (2024) developed a cross-attention-based fusion network for distinguishing Parkinsonian tremor from essential tremor, achieving high performance [54]. Similarly, Sun et al. (2024) introduced CMAF-Net for incomplete multimodal brain tumour segmentation, showing that cross-attention outperformed conventional fusion strategies [55].

By contrast, the three alternative fusion strategies we tested (simple CONCAT, weighted sum, and dual-stream) all yielded poor performance. Similar findings have been reported in related multimodal studies. For example, Zhao et al. (2025) introduced AMFI-Net, an attention-based multimodal fusion framework, and provided empirical evidence that attention-based fusion consistently outperformed traditional integration methods such as concatenation and average across diverse classification and recognition tasks [56]. Taken together, cross-attention is required to achieve robust performance in clinical multimodal settings. This may be because cross-attention provides a powerful mechanism for aligning heterogeneous modalities; its primary advantage lies in its ability to selectively weight informative features across domains, thereby substantially improving predictive accuracy [54,55]. In addition, cross-attention enhances robustness by mitigating modality imbalance and noise sensitivity, enabling the model to exploit complementary information more effectively than naive fusion strategies [57,58].

The strength of recent study, first, our choice to encode time series as images is supported by growing theory and practice showing that GAF preserves temporal dependencies in a 2D Hamiltonian-like Gramian while exposing patterns to mature vision backbones; recent applications in sensing and forecasting report accuracy gains versus 1D modelling, and new variants further emphasise the representational value of angle-based embeddings [20,21,39]. Second, our cross-attention fusion operationalises a form of learnable query, key, and value interaction between clinical and movement features [59,60], consistent with the direction of modern multimodal medical AI [61], where attention replaces static concatenation to capture complementary cues and suppress noise better [62]. Third, our findings contribute to the literature on wearable-based ADHD. Recent feasibility and modelling studies using sensor-based measurement of physical activity metrics can discriminate ADHD phenotypes and augment questionnaire-based assessment, underscoring the clinical promise and ecological validity of free-living measurement [63,64].

Despite the promising classification performance, several limitations should be acknowledged. First, although the study included a comparative evaluation of fusion strategies where the proposed cross-attention mechanism clearly outperformed simple concatenation, weighted sum, and dual-stream methods, the analysis did not extend to comparisons involving alternative backbone architectures for activity image recognition. The current pipeline relies on a ViT-BiLSTM encoder for temporal–spatial representation, but its relative advantage over other potential models was not systematically examined. Including such comparisons in future work would help clarify whether the observed performance gains arise mainly from the fusion design or the specific feature encoder employed.

Second, although several strategies were applied to mitigate class imbalance, potential bias may still persist due to the limited dataset size and unequal class distribution. Expanding the dataset and incorporating additional data augmentation or transfer learning techniques would help enhance robustness and reduce bias in future studies.

Lastly, although the Apple Watch used in this study has been validated in previous research for measuring physical activity, the number of validation studies remains limited, and its application in model-based prediction tasks is still relatively scarce. Further studies are therefore needed to examine its robustness across diverse contexts and populations, so as to ensure the reliability and consistency of wearable-derived signals in predictive modelling.

Future work should therefore incorporate post hoc explanation techniques (e.g., Grad-CAM for ViT tokens, SHAP for tabular branches) to provide transparent insights into which activity or clinical features most strongly contribute to predictions. Moreover, wearable sensor data are inherently susceptible to label noise (e.g., questionnaire thresholds, contextual biases) and class imbalance, which highlights the importance of calibrated decision thresholds, cost-sensitive learning, and uncertainty estimation in real-world applications [65].

## 5. Conclusions

We introduced the Acceleration Transformer Multimodal Network (VB-Multimodal), which reparameterises wrist sensor as GAF images, encodes spatial, temporal dynamics with a ViT-BiLSTM branch, and fuses these with clinical features via cross-attention. This design consistently surpasses standard late-fusion baselines and captures clinically meaningful signals, indicating real value for naturalistic ADHD screening. Remaining gaps include single-site scope and device/domain variability. Future work should prioritise external validation across devices and populations, large-scale, trustworthy interpretability, uncertainty estimation, and efficient on-device deployment for privacy-preserving use.

## Figures and Tables

**Table 1:** Overview of multimodal network applications for ADHD prediction.

References	Dataset	Data Type	Arch/Model	Validation Metrics	Validation Accuracy
1. O’Mahony et al. (2014) [28]	Private 6–11 years (*n* = 43, 26 male)	Tabular	SVM	Accuracy; Sensitivity; Specificity;	SVM = 94%
2. Faedda et al. (2016) [27]	Private 5–18 years (*n* = 155, 97 male)	Tabular	RF; ANNs; PLS; MR; SVM	Accuracy; Kappa; ROC-AUC	RF = 79%; ANNs = 81%; PLS = 82%; MR = 82%; SVM = 83%
3. Duda et al. (2017) [31]	Private 10–14 years (*n* = 174, 93 male)	Tabular	SVM; Ridge; ENet; LDA	ROC-AUC	SVM = 78%; Ridge = 84%; Enet = 89%; LDA = 89%
4. Muñoz-Organero et al. (2018) [32]	Private 6–15 years (*n* = 22, 11 male)	Image	CNN	Accuracy; Sensitivity; Specificity;	CNN = 93%
5. Kim et al. (2023) [11]	ABCD dataset 9-Dataset 11 years (*n* = 1090, 513 male)	Tabular	RF; XGB; LGB	AUC; Sensitivity; Specificity;	RF = 73%; XGB = 78%; LGB = 79%
6. Park et al. (2023) [3]	Private 7–16 years (*n* = 39, 31 male)	Tabular	RF	Accuracy; Recall; Precision; F1; AUC	RF = 78%;
7. Rahman (2024) [29]	ABCD dataset 9-Dataset 11 years (*n* = 450, 257 male)	Tabular	RF; Ada; DT; KNN; LGBM; LR; NB; SVM	Accuracy; Recall; Precision; F1;	RF = 87%; Ada = 65%; DT; KNN = 53; LGBM = 74%; LR = 61%; NB = 58%; SVM = 53%
8. Jiang et al. (2024) [30]	Private 16–17 years (*n* = 30, 16 male)	Tabular	XGBoost	Accuracy; Recall; Precision; F1;	XGBoost = 93%

Note: RF: random forest; ANNs: artificial neural networks; PLS: partial least squares; MR: multinomial regression; SVM: support vector machine; Ridge: ridge regression; LDA: linear discriminant analysis; ENet: elastic net; CNN: convolutional neural network; ROC-AUC: area under the receiver operating characteristic curve; ABCD dataset: Adolescent Brain Cognitive Development in US.

**Table 2:** Descriptive characteristics of the study participants (n = 50).

Characteristic	Total (n = 50)	Male (n = 29)	Female (n = 21)	Difference *p*
Age, years (Mean ± SD)	9.16 ± 1.68	9.17 ± 1.79	9.14 ± 1.56	0.95
BMI (Mean ± SD)	16.57 ± 2.69	16.93 ± 2.95	16.07 ± 2.25	0.24
Grade (n, %)				0.61
Grade 1	10 (20.0)	6 (20.7)	4 (19.0)	
Grade 2	13 (26.0)	7 (24.1)	6 (28.6)	
Grade 3	11 (22.0)	6 (20.7)	5 (23.8)	
Grade 4	11 (22.0)	6 (20.7)	5 (23.8)	
Grade 5	2 (4.0)	2 (6.9)	0 (0.0)	
Grade 6	2 (4.0)	2 (6.9)	0 (0.0)	
Grade 7	1 (2.0)	0 (0.0)	1 (4.8)	
Socioeconomic factors (n, %)				
Health state				0.89
Poor	7 (14.0)	4 (13.8)	3 (14.3)	
Good	43 (86.0)	25 (86.2)	18 (85.7)	
Very Good	0 (0.0)	0 (0.0)	0 (0.0)	
Diet state				0.56
Poor	13 (26.0)	6 (20.7)	7 (33.3)	
Good	35 (70.0)	22 (75.9)	13 (61.9)	
Very Good	2 (4.0)	1 (3.4)	1 (4.8)	
Parental education (n, %)				0.94
Poor	8 (16.0)	5 (17.2)	3 (14.3)	
Moderate	40 (80.0)	23 (79.3)	17 (81.0)	
Good	2 (4.0)	1 (3.4)	1 (4.8)	
SNAP-IV scores (Mean ± SD)				
Inattention score	15.76 ± 4.11	16.93 ± 4.02	14.14 ± 3.76	0.01
Hyperactivity score	13.80 ± 3.42	14.72 ± 3.39	12.52 ± 3.09	0.02
Oppositional score	13.60 ± 2.60	14.24 ± 2.84	12.71 ± 1.95	0.02
SNAP-IV Total	42.98 ± 9.13	45.69 ± 9.23	39.24 ± 7.72	0.01
ADHD classification				
Non-ADHD (T < 55)	37 (74.0)	18 (62.1)	19 (90.5)	0.04
ADHD (T ≥ 55)	13 (26.0)	11 (37.9)	2 (9.5)	0.02

Note: Values are presented as mean ± SD for continuous variables and n (%) for categorical variables. Grade refers to the school grade level of the participants. Socioeconomic factors (health state, diet state, and parental education) were assessed on a 3-point scale (1 = poor/low, 2 = moderate/average, 3 = good/high). SNAP-IV: Swanson, Nolan, and Pelham Questionnaire; ADHD: attention-deficit/hyperactivity disorder. Differences in *p* values represent comparisons between males and females, calculated using independent *t*-tests for continuous variables and chi-square or Fisher’s exact tests for categorical variables, as appropriate.

**Table 3:** Performance of VB-Multimodal.

	Precision	Recall	F1	Accuracy	AUC	MCC
VB-Multimodal	0.97	0.97	0.97	0.97	0.99	0.93

**Table 4:** Classification performance comparison of different fusion methods in VB-Multimodal.

Fusions	Precision	Recall	F1	Accuracy	AUC	MCC
Cross-attention	0.97	0.97	0.97	0.97	0.99	0.93
Simple CONCAT	0.42	0.35	0.37	0.35	0.29	−0.32
Weightedsum	0.48	0.35	0.34	0.35	0.45	−0.14
Dual-stream	0.58	0.46	0.47	0.46	0.35	0.02

## Data Availability

The multimodal dataset used in this study, including accelerometer data and structured clinical variables, has been publicly released (https://doi.org/10.5281/zenodo.14875672). The model code of this study will be made publicly available after acceptance of the manuscript.

## Abbreviations

The following abbreviations are used in this manuscript:

ADHD	Attention-Deficit/Hyperactivity Disorder
GAF	Gramian Angular Field
ENMO	Euclidean Norm Minus One
ViT	Vision Transformer
BiLSTM	Bidirectional Long Short-Term Memory
EEG	Electroencephalography
SNAP-IV	Swanson, Nolan, and Pelham Questionnaire, 4th Edition
SD	Standard Deviation
BMI	Body Mass Index
SVM	Support Vector Machine
RF	Random Forest
XGB	XGBoost
LGB	Light Gradient Boosting Machine (LightGBM)
ANNs	Artificial Neural Networks
CNN	Convolutional Neural Network
ROC-AUC	Receiver Operating Characteristic–Area Under the Curve
